# Dr. George J. Barclay

**Published:** 1878-04

**Authors:** 


### OBITUARY.

---

*Dr. George J. Barclay,* of Pittsburgh, Pa., died suddenly of internal hemorrhage, at the residence of Dr. W. H. Atkinson, New York City, on the morning of the twenty-second of January 1878, in the fifty-third year of his age.

Dr. Barclay was a superior operator, and unusually courteous with his patients. The dental profession has few such members to lose.

